# Efficacy of prophylactic tranexamic acid in preventing postpartum hemorrhage following repeat cesarean sections: a retrospective matched-cohort study

**DOI:** 10.3389/fdsfr.2026.1911287

**Published:** 2026-10-16

**Authors:** Xiaodan Di, Xiaojun Li, Zheng Zheng, Lanlan Guo, Taowei Dong, Qiaozhu Chen

**Affiliations:** Guangzhou Women and Children’s Medical Center, Guangzhou Medical University, Guangzhou, China

**Keywords:** Postpartum Hemorrhage, propensity score matching, repeat cesarean section, retrospective cohort study, tranexamic acid

## Abstract

**Background:**

Postpartum hemorrhage (PPH) is a leading cause of maternal mortality, and women with repeat cesarean sections are at high risk. This study aimed to evaluate the efficacy of prophylactic tranexamic acid (TXA) in preventing PPH among women undergoing repeat cesarean sections.

**Methods:**

A retrospective cohort study was conducted including women with repeated cesarean sections at a tertiary obstetrical center in South China between July 2016 and December 2023. Patients were divided into the TXA group and the control group according to intraoperative prophylactic TXA administration. Given the imbalance in baseline characteristics, 1:2 propensity score matching (PSM) with a caliper of 0.01 was performed to balance confounding variables. The primary outcome was PPH incidence. Secondary outcomes included estimated blood loss, hemoglobin decline, additional uterotonic use, and blood transfusion rate.

**Results:**

After 1:2 PSM, 2,777 women in the TXA group were matched with 5,554 women in the control group, with baseline characteristics well balanced. The incidence of PPH was significantly lower in the TXA group than in the control group (11.09% vs. 17.03%, P < 0.001). The TXA group also demonstrated less calculated estimated blood loss, smaller hemoglobin reduction (12.06% vs. 18.44%, P < 0.001), and lower requirements for additional uterotonic agents (26.90% vs. 29.80%, P = 0.006). Multivariate logistic regression confirmed that prophylactic TXA was an independent protective factor against PPH (OR 0.620, 95% CI 0.540-0.712, P < 0.001). No significant difference was observed in blood transfusion rates (1.55% vs. 2.09%, P = 0.089).

**Conclusion:**

Prophylactic TXA use was associated with a lower incidence of PPH and the need for additional uterotonics in women undergoing repeat cesarean sections. Randomized trials in this specific population are needed.

## 1 Introduction

Postpartum hemorrhage (PPH) is a major cause of maternal mortality globally, particularly in resource-poor regions. In the 20th century, approximately 28% of maternal deaths reported in sub-Saharan Africa and South Asia were attributed to PPH (Musarandega et al., 2021). Repeat cesarean section is a significant contributor to PPH, with studies demonstrating that the risk is related to the times of repeat cesarean section, if more than 5 times the risk will be 1.8 times higher (Ajmal et al., 2024); and another study shows that the peritoneal adhesions during cesarean delivery are an independent risk factor for peri-partum hemorrhagic complications (Saban et al., 2020).

Tranexamic acid (TXA) is a commonly employed antifibrinolytic agent, used to prevent and treat bleeding in various surgical and traumatic scenarios (UK Royal Colleges Tranexamic Acid in Surgery Implementation Group et al., 2022; Shakur et al., 2010; CRASH-3 Trial Collaborators, 2019). There is sufficient evidence to confirm that TXA effectively reduces blood loss and mortality in procedures like cesarean section (CS) and in post-traumatic hemorrhage management (Cheema et al., 2023; Obstetrics Subgroup et al., 2023a). Its use in obstetrics has been verified, which can lower the mortality of PPH (Sentilhes et al., 2018).

Therefore, the World Health Organization recommends early intravenous injection of TXA for patients with confirmed PPH (World Health Organization, 2017). However, the evidence for the prophylactic use of TXA during cesarean section is complex and evolving. While numerous small-to-medium-sized randomized controlled trials (RCTs) and subsequent meta-analyses have suggested that TXA reduces blood loss and transfusion requirements, the conclusions from these syntheses are limited by the overall low quality and high risk of bias in the included studies (Cheema et al., 2023; Rohwer et al., 2024). Crucially, recent large-scale, high-quality RCTs have yielded mixed or null findings regarding clinically significant outcomes. The multicenter, double-blind, randomized, controlled trial found that while TXA reduced a composite outcome of calculated blood loss >1000 mL or red-cell transfusion, it did not significantly reduce the need for additional uterotonics or other clinically relevant hemorrhagic morbidity (Sentilhes et al., 2021). Similarly, another major RCT found no significant reduction in a composite of hemorrhage-related outcomes with prophylactic TXA (Pacheco et al., 2023). Furthermore, the optimal candidate population for prophylaxis remains undefined. Women undergoing repeat cesarean sections constitute a recognized high-risk subgroup for PPH (Saban et al., 2020), yet they have not been the specific focus of large RCTs. To date, no large-scale study has specifically evaluated TXA prophylaxis in women with prior uterine scars. Therefore, this retrospective cohort study was designed to evaluate the effectiveness of TXA prophylaxis specifically among women undergoing repeat cesarean sections, aiming to provide hypothesis-generating evidence for this high-risk population.

## 2 Materials and methods

### 2.1 Study design

The study was a retrospective cohort study conducted at a tertiary obstetrical center (Guangzhou Women and Children’s Medical Center, Guangzhou Medical University [GWCMC]). All pregnant women who underwent repeated cesarean sections between July 1, 2016, and December 31, 2023 were initially enrolled.

### 2.2 Inclusion criteria

Parous women who had undergone repeat cesarean section because of uterine scar during the study period. Women with uterine scars due to other factors (like previous myomectomy) were excluded. The inclusion criteria were as follows: (1) singleton pregnancy; (2) repeated cesarean section (The number of previous cesarean sections is ≥ 1); (3) complete medical records and perioperative data.

### 2.3 Exclusion criteria

Exclusion criteria included anemia (hemoglobin <80 g/L), maternal comorbidities (e.g., heart disease, renal disease, liver disease, or coagulation disorders), a history of thromboembolic disease, or known allergy to tranexamic acid. Individuals presenting a high risk for obstetric hemorrhage, including those with conditions like placenta previa, placenta accreta, placental abruption, or uterine rupture, were excluded from the study.

### 2.4 Grouping and intervention

According to whether prophylactic tranexamic acid was administered intraoperatively, eligible patients were divided into two groups: the tranexamic acid group and the control group. The dosage and administration route of tranexamic acid followed routine clinical protocols. Administration was completed within 60 min after delivery, with a 1 g intravenous infusion over 10 min after cord clamping. Because of the retrospective design, we could not reliably differentiate between prophylactic use and therapeutic use for acute PPH. We considered that it was not appropriate to just assume that all women diagnosed with PPH received TXA, since real-time visual estimation usually underestimates actual blood loss and has limited sensitivity for identifying PPH (American College of Obstetricians and Gynecologists, 2019). Although the decision to administer prophylactic TXA was based on the attending obstetrician’s clinical judgment considering individual patient risk factors, our center did not have a mandatory, standardized protocol for its use during the study period. This practice pattern was consistent throughout the study period, and we did not observe a significant temporal trend in TXA usage (data not shown).

### 2.5 Propensity score matching (PSM)

To minimize selection bias and confounding due to imbalanced baseline characteristics between the two groups, 1:2 propensity score matching was performed. A logistic regression model was used to calculate propensity scores, with TXA administration as the dependent variable. The following covariates were included based on their known association with PPH and the risk of imbalance between groups: maternal age (continuous), pre-pregnancy BMI (continuous), gestational age (continuous), general anesthesia (binary), gestational diabetes mellitus (binary), gestational hypertensive disorders (binary), neonatal birth weight (continuous), and macrosomia (binary, birth weight >4000 g). The nearest-neighbor matching method with a caliper of 0.01 was applied. After PSM, baseline balance was verified using standardized mean differences (SMD < 0.10).

### 2.6 Outcome measures

The primary outcome was PPH, defined as an estimated blood loss (EBL) ≥1000 mL within 24 h after cesarean section or red blood cell transfusion within 2 days after delivery. Secondary outcome measures include blood product transfusion, absolute hemoglobin difference (g/L), and other clinical interventions related to hemorrhage management (like uterine artery embolization, additional uterotonic agents such as intramuscular injection of 1 mL [250 μg] carboprost tromethamine), as well as any maternal thromboembolic events.

Calculated EBL was estimated using the formula: EBL = EBV × (preoperative Hct–postoperative Hct)/preoperative Hct. Preoperative Hct was defined as the most recent value within 8 days before delivery. For the primary analysis of calculated EBL, the postoperative Hct was defined as the value measured on the second day after delivery (POD 2) in patients who did not receive any blood transfusion. Patients who received a transfusion were excluded from this specific secondary analysis of calculated EBL, as their Hct values would be artificially altered by the transfusion. However, they were included in the primary outcome of PPH (which includes transfusion) and the analysis of blood transfusion rates. This approach is consistent with the methodology used in the TRAAP2 trial (Sentilhes et al., 2021). This EBL estimation method was chosen because the subjective EBL estimation during cesarean section is limited (American College of Obstetricians and Gynecologists, 2019).

While calculated EBL using the hematocrit-based formula is a more objective measure than visual estimation, it is subject to influence from perioperative fluid administration, postoperative hemodilution, and the timing of hematocrit measurements. We used a standardized protocol at our institution and the earliest postoperative hematocrit (POD 2) to minimize this variability. The baseline characteristics of groups were balanced after matching, mitigating the effect of different fluid management strategies.

### 2.7 Data collection

Clinical data were extracted from electronic medical records. The primary outcome was the incidence of postpartum hemorrhage (PPH). Secondary outcomes included estimated blood loss, hemoglobin drop, the need for additional uterotonic agents, blood transfusion rate, etc.

### 2.8 Analysis of statistical data

Continuous variables were presented as mean ± standard deviation (SD) or median (interquartile range, IQR) and compared using the Student’s t-test or Mann-Whitney U test. Categorical variables were expressed as numbers and percentages (%) and analyzed using the chi-square test or Fisher’s exact test. To eliminate selection bias in the use of TXA, the propensity scores for TXA administration were determined by the known risk factors for PPH, such as gestational hypertension, gestational diabetes mellitus, gestational age, and neonatal birth weight, which were used as predictive variables. Each woman who received TXA was matched with two women who did not receive TXA by using the nearest available propensity score. After the matching was done, the standardized mean differences (SMD) were evaluated to guarantee balance. Logistic regression analysis was employed to assess the relationship between TXA administration and the primary outcome. All analyses utilized SPSS software, version 26.0. A two-sided P-value < 0.05 was considered statistically significant.

### 2.9 Ethics

Given the retrospective nature of the study, informed consent was not necessary. The study was carried out in line with the STROBE guidelines (Ajmal et al., 2024). Ethical approval was obtained from the Institutional Review Board of GWCMC (Approval No.: Suifu’erke Lunpi Zi [2025] No.493A01).

## 3 Results

During the study period, 20,723 patients were subjected to cesarean section because of uterine scar. After excluding 575 patients with uterine scars from non-cesarean surgeries, 1145 patients with gestational age <37 weeks, and patients with high-risk factors for severe PPH (placenta accreta, placenta previa, placental abruption, uterine rupture), 18,437 parous women who underwent repeat cesarean section were initially enrolled. After further excluding 1365 cases with incomplete data, 73 cases with preoperative hemoglobin <80 g/L, 1 case aged <18 years, and 46 non-Chinese nationals, a total of 16,952 cases were finally included in the study cohort (n = 16,952) (Figure 1). Of the total participants, TXA treatment was administered to 2,777 women, accounting for 16.38%. There were a total of 1678 cases of PPH, with an incidence rate of 9.90%. A total of 187 cases had a postpartum hemoglobin drop of more than 20 g/L. For detailed information, please refer to Figure 1.

**FIGURE 1:**
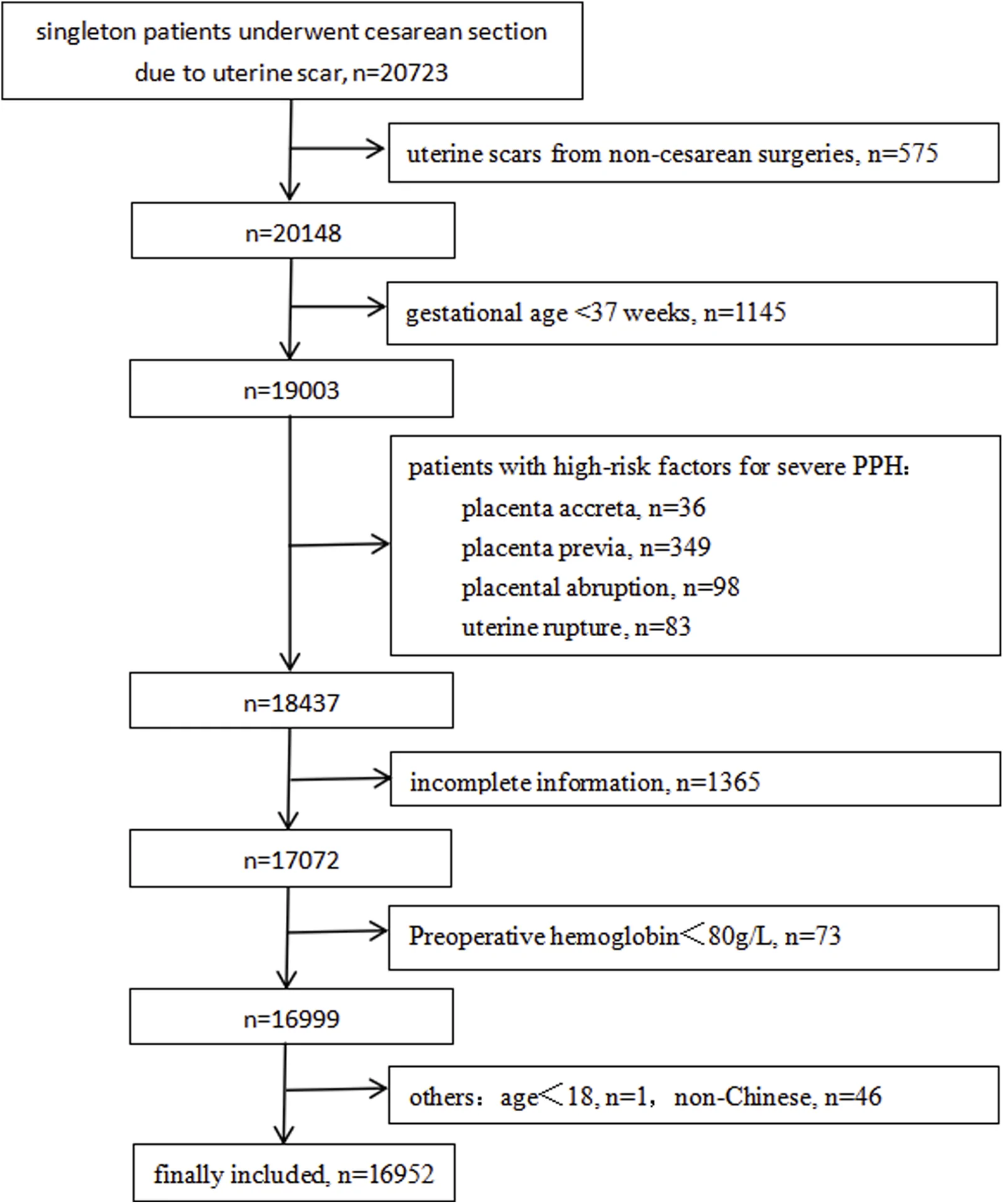
Flowchart of the population included in the study.

### 3.1 Baseline characteristics before and after PSM

Before PSM, a total of 16,952 patients were included in the study, with 2,777 in the TXA group and 14,175 in the control group. After 1:2 propensity score matching, slight intergroup differences remained in maternal BMI, gestational age, and birth weight (all P < 0.05). These variables were considered weak confounding factors for postpartum hemorrhage, and their imbalances were mild. To further eliminate residual confounding and ensure the robustness of the results, multivariate logistic regression analysis was used to adjust for these variables in the subsequent analysis. All other baseline characteristics achieved good balance after PSM (all P > 0.05). (Table 1).

**TABLE 1:** Baseline characteristics of study participants before and after propensity score matching (n = 16,952).

Characteristic	Before matching			After matching		
	No TXA (n = 14,175)	TXA (n = 2777)	P value	No TXA (n = 5554)	TXA (n = 2777)	P value
Maternal age (years, mean ± SD)	33.89 ± 4.06	33.84 ± 4.05	0.51	33.90 ± 4.06	33.84 ± 4.05	0.505
BMI(Body-mass index before pregnancy) (mean ± SD)	27.05 ± 3.14	27.04 ± 3.14	0.931	27.09 ± 1.52	27.04 ± 3.14	0.512
Gestational age (weeks ± SD)	38.53 ± 0.74	38.48 ± 0.75	0.001	38.48 ± 0.36	38.48 ± 0.75	0.875
General anesthesia (n. %)	191 (1.35)	18 (0.65)	0.002	49 (0.88)	18 (0.65)	0.260
Gestational diabetes (n. %)	3282 (23.15)	640 (23.05)	0.903	1334 (24.02)	640 (23.05)	0.325
Gestational hypertensive disorders (n. %)	542 (3.82)	145 (5.22)	<0.001	240 (4.32)	145 (5.22)	0.065
Neonatal sex (male) (n. %)	7517 (53.03)	1510 (54.38)	0.194	2951 (53.13%)	1510 (54.38)	0.284
Birthweight (g, mean ± SD)	3286.22 ± 372.23	3278.24 ± 385.17	0.304	3283.15 ± 376.60	3278.24 ± 385.17	0.583
Macrosomia (>4000 g) n (%)	490 (3.46)	102 (3.67)	0.57	233 (4.20)	102 (3.67)	0.253
Apgar score at 1 min ≤ 7 n (%)	22 (0.16)	4 (0.14)	1	11 (0.20)	4 (0.14)	0.584

TXA: tranexamic acid; PPH: postpartum hemorrhage.

Data are presented as mean ± standard deviation (SD) for continuous variables and number (percentage) for categorical variables.

P values were calculated using Student’s t-test for continuous variables and chi-square test for categorical variables.

### 3.2 Matching based on propensity scores

Stratified analysis based on TXA use showed that the incidence of PPH was significantly higher in the TXA group than in the non-TXA group (308 cases, 11.09% vs. 1370 cases, 9.66%; P = 0.021) before PSM. To mitigate selection bias, we employed a 1:2 nearest neighbor propensity score matching (PSM) approach, utilizing a caliper width set at 0.01 times the standard deviation of the propensity score. The matching process incorporated several covariates, namely, maternal age, pre-pregnancy body mass index (BMI), gestational age, use of general anesthesia, presence of gestational diabetes mellitus, gestational hypertensive disorders, and neonatal birth weight. Finally, in the TXA group, there were 2,777 cases matched with 5,554 cases in the non-TXA group, resulting in a total sample size of 8331 after the matching. The SMD of all covariates was <0.1, indicating a good baseline balance. In the matched cohort, there were 1,254 cases of PPH (15.05% of the 8,331 matched patients).

Matched analysis showed that the incidence of PPH in the TXA group (308, 11.09%) was significantly lower than that in the non-TXA group (946, 17.03%) (P < 0.001). For secondary outcomes, the blood transfusion rate was 43 (1.55%) in the TXA group and 116 (2.09%) in the non-TXA group (P = 0.089), and the rate of additional carboprost tromethamine use was 747 (26.9%) in the TXA group and 1655 (29.8%) in the non-TXA group (P = 0.006), as shown in Table 2.

**TABLE 2:** Comparison of primary and secondary outcomes between TXA and Non-TXA groups after propensity score matching.

Outcome	No TXA (n = 5554)	TXA (n = 2777)	P value	t/Chi-square
Postpartum hemorrhage (no. %)	946 (17.03)	308 (11.09)	<0.001	51.115
Blood transfusion	116 (2.09)	43 (1.55)	0.089	2.885
Hb drop≥ 20 g/L (no. %)	1024 (18.44)	335 (12.06)	<0.001	55.093
Hematocrit peripartum decrease ≥10% (no. %)	133 (2.39)	33 (1.19)	<0.001	13.796
Additional uterotonic agents for excessive bleeding (carboprost tromethamine) (no. %)	1655 (29.8)	747 (26.9)	0.006	7.582
Arterial embolization, emergency surgery for postpartum hemorrhage, or artery ligation, or hysterectomy (no. %)	10 (0.18)	14 (0.5)	0.009	6.770
Calculated estimated blood loss category (no. %)				
>500 mL	2750 (49.51)	1072 (38.6)	<0.001	88.765
>1000 mL	873 (15.72)	276 (9.94)	<0.001	52.013

### 3.3 Multivariate logistic regression analysis

Further multivariate logistic regression analysis showed that after adjusting for age, gestational age, BMI, general anesthesia, gestational hypertensive disorders, gestational diabetes mellitus, and macrosomia, prophylactic use of TXA was an independent protective factor for PPH after repeat cesarean section (OR = 0.620, 95% CI [0.540-0.712], P < 0.001) (Table 3).

**TABLE 3:** Multivariate logistic regression analysis for PPH.

Variable	OR	95%CI	P-value
TXA	0.620	0.540-0.712	<0.001
Age	0.987	0.972-1.002	0.087
Gestational age	0.963	0.885-1.048	0.379
BMI	0.940	0.922-0.958	<0.001
General anesthesia	0.561	0.318-0.988	0.045
Gestational hypertensive disorders	0.760	0.582-0.994	0.045
Gestational diabetes mellitus	0.900	0.781-1.036	0.141
Macrosomia	0.877	0.640-1.201	0.412

## 4 Discussion

This large, real-world retrospective cohort study, employing rigorous propensity score matching (PSM) to mitigate selection bias, provides evidence that prophylactic administration of 1 g tranexamic acid (TXA) during repeat cesarean section is associated with a significant reduction in the incidence of postpartum hemorrhage (PPH), calculated blood loss, and the need for additional uterotonic agents. Multivariate analysis confirmed TXA as an independent protective factor (OR 0.620, 95% CI 0.540-0.712). These findings offer valuable, targeted evidence for a high-risk obstetric population that has been underrepresented in large-scale randomized trials.

### 4.1 Comparison with existing evidence and contextualization

Our results align with the conclusions of several meta-analyses of randomized controlled trials (RCTs) that reported TXA’s efficacy in reducing blood loss during cesarean delivery (Cheema et al., 2023). However, it is critical to interpret these meta-analyses with caution. The most recent and comprehensive meta-analysis, while showing a reduction in blood loss >1000 mL and transfusion needs, explicitly graded the certainty of this evidence as low, citing serious limitations in the design and reporting of many included trials (Cheema et al., 2023). This underscores that the apparent consensus from pooled small studies may not reflect definitive, high-quality evidence.

The most direct challenge to the routine prophylactic use of TXA comes from recent large-scale, pragmatic RCTs. The TRAAP2 trial, a high-quality study involving over 11,000 women undergoing cesarean delivery, found that TXA did not lead to a significant reduction in the use of additional uterotonic agents—a key clinical indicator of hemorrhage—nor in provider-assessed clinically significant hemorrhage (Sentilhes et al., 2021). Our study, focused on the repeat cesarean subgroup, found a reduction in both calculated blood loss and additional uterotonic use. Several factors may explain this apparent discrepancy.

First, population specificity is paramount. Women with prior uterine scars face a heightened risk from both uterine atony and surgical wound oozing (Ajmal et al., 2024). TXA’s antifibrinolytic action may be particularly effective against the latter mechanism, potentially yielding a greater relative benefit in this subgroup than in an unselected cesarean population. This hypothesis is supported by a small RCT specifically in elective repeat cesarean deliveries, which also suggested a benefit (Ogunkua et al., 2022). Second, outcome measurement plays a role. Our primary outcome relied on calculated estimated blood loss, a method also used in TRAAP2’s primary outcome. TRAAP2 showed TXA was effective for this calculated metric but not for clinically driven outcomes. Our study similarly shows improvement in calculated blood loss categories. This highlights an important question: whether reduction in calculated blood loss reliably translates to meaningful clinical benefit. Third, study design differences are inherent. As a retrospective observational study, despite rigorous matching, residual confounding (e.g., unmeasured surgeon preference, subtle indications for TXA use) cannot be entirely ruled out, whereas RCTs like TRAAP2 provide the highest level of evidence by design.

The reliance on calculated EBL as a primary endpoint, as opposed to a composite of clinically meaningful outcomes, is a limitation shared with some large RCTs (e.g., TRAAP2). The reduction in calculated EBL may not always translate into a reduction in clinically driven interventions like transfusion, a finding also observed in our study (Table 2). Future studies in this high-risk population should prioritize clinically relevant outcomes, such as the need for advanced uterotonics or composite of severe maternal morbidity.

### 4.2 Clinical implications and interpretation of secondary outcomes

The significant reduction in the need for additional uterotonic agents (carboprost tromethamine) in the TXA group (26.9% vs. 29.8%, P = 0.006) is a clinically relevant finding. It suggests that TXA may reduce the reliance on second-line, more potent uterotonics, which are associated with greater side effects. This synergistic effect between TXA and oxytocin is biologically plausible and supports the role of TXA as an adjunctive hemostatic agent.

The lack of a statistically significant difference in blood transfusion rates (1.55% vs. 2.09%, P = 0.089) warrants discussion. This may be attributable to our center’s strict adherence to restrictive transfusion thresholds (hemoglobin <70 g/L or symptomatic anemia) as per national guidelines (Obstetrics Subgroup et al., 2023b), which minimizes unnecessary transfusions and thus reduces the power to detect a difference. This finding is consistent with other studies where transfusion was not a primary driver of the treatment effect (Sentilhes et al., 2021).

Interestingly, we observed a significantly higher rate of severe hemorrhage-related interventions (arterial embolization, hysterectomy, or emergency surgery) in the TXA group (0.5% vs. 0.18%, P = 0.009). This finding is counterintuitive given the overall lower incidence of PPH in the TXA group. We propose several potential explanations for this observation. First, this finding may be a statistical artifact due to the very small number of events (N = 14 vs. N = 10), which can lead to unstable estimates. Second, it is possible that when PPH did occur in the TXA group, it was more severe or refractory to medical management, leading to a higher rate of surgical intervention. However, given the small numbers, we cannot draw any firm conclusions. Third, it is possible that the clinicians’ threshold for surgical intervention differed between groups, though this is speculative. We have added this as a limitation and emphasize that this finding should be considered hypothesis-generating and requires further investigation in larger, prospective studies.

### 4.3 Safety considerations

We observed no thromboembolic events in our cohort, consistent with the short-term safety profile reported in many studies (Shakur et al., 2010). While this is reassuring, we must caution that the retrospective nature of this study, the lack of active surveillance for thromboembolism, and the relatively low incidence of these events in the general obstetric population, limit our ability to draw definitive conclusions regarding the safety of prophylactic TXA. The small numerical increase in thromboembolic events observed in the TRAAP2 trial (Sentilhes et al., 2021) highlights the need for ongoing vigilance and prospective safety data collection, specifically in this high-risk subgroup. However, it is essential to note that large trials have reported increased incidences of non-thromboembolic adverse effects (e.g., nausea, vomiting) with TXA (Sentilhes et al., 2018; Sentilhes et al., 2021), which our retrospective data did not capture. Therefore, the long-term and large-scale safety profile, especially in high-risk obstetric populations, requires continued vigilance and evaluation in prospective studies.

### 4.4 Strengths and limitations

This study has several notable strengths. First, its large sample size (n = 16,952) from a single tertiary center provides robust real-world data. Second, we rigorously excluded women with major placental abnormalities (placenta previa, accreta, abruption) to reduce confounding from these extreme bleeding risks. Third, the use of 1:2 PSM and multivariate logistic regression effectively balanced baseline characteristics and adjusted for residual confounders. Fourth, we employed a calculated blood loss estimate, which is more objective than visual estimation (American College of Obstetricians and Gynecologists, 2019).

The limitations are inherent to the retrospective design. First, selection bias cannot be entirely eliminated despite statistical adjustments; unmeasured confounders such as surgeon experience or specific intraoperative findings may have influenced both TXA administration and outcomes. Second, we lacked detailed data on the exact timing of TXA infusion (e.g., pre-incision vs. post-delivery) and could not analyze dose-response relationships. Third, being a single-center study, our findings may be influenced by local protocols and practices, limiting generalizability. Fourth, the generalizability of our findings is limited to a relatively selected population of women undergoing repeat cesarean section without major placental abnormalities or severe anemia. This deliberate exclusion was to reduce confounding from these extreme bleeding risks, thereby improving the internal validity of the study. However, clinicians should not extrapolate these results to women with conditions like placenta previa or accreta, who represent a distinct and much higher-risk group for PPH. Future research should specifically target these high-risk subgroups. Fifth, we did not assess longer-term maternal outcomes such as postpartum anemia recovery, duration of hospital stay, or uterine scar healing in subsequent pregnancies. As a retrospective study, the non-randomized assignment of TXA introduced unavoidable selection bias. Despite rigorous PSM and multivariate adjustment, unmeasured confounders—such as surgeon preference, intraoperative findings of dense adhesions, or subtle clinical signs of early bleeding—may have influenced both the decision to administer TXA and the subsequent outcomes. Therefore, causal inference should be made with caution. And, the calculated EBL as a primary endpoint has limitations. In clinical practice, it is still necessary to focus on the occurrence of severe maternal complications.

### 4.5 Conclusion and future directions

In conclusion, in this high-risk subgroup of women undergoing repeated cesarean sections, prophylactic TXA was associated with a reduction in calculated blood loss and the need for additional uterotonics. These hypothesis-generating findings warrant confirmation in a dedicated randomized controlled trial.

## Data Availability

The datasets presented in this article are not readily available because due to patient consent restrictions, the dataset is not publicly available. Requests to access the datasets should be directed to XD, dxd_333@163.com.
